# Long-Term Tracking of Group-Housed Livestock Using Keypoint Detection and MAP Estimation for Individual Animal Identification

**DOI:** 10.3390/s20133670

**Published:** 2020-06-30

**Authors:** Eric T. Psota, Ty Schmidt, Benny Mote, Lance C. Pérez

**Affiliations:** 1 Department of Electrical and Computer Engineering, University of Nebraska–Lincoln, Lincoln, NE 68505, USA; lperez@unl.edu; 2 Department of Animal Science, University of Nebraska–Lincoln, Lincoln, NE 68588, USA; ty.schmidt@unl.edu (T.S.); benny.mote@unl.edu (B.M.)

**Keywords:** precision livestock, multi-object tracking, keypoint detection, activity tracking, long-term tracking, animal behavior, maximum a posteriori classification

## Abstract

Tracking individual animals in a group setting is a exigent task for computer vision and animal science researchers. When the objective is months of uninterrupted tracking and the targeted animals lack discernible differences in their physical characteristics, this task introduces significant challenges. To address these challenges, a probabilistic tracking-by-detection method is proposed. The tracking method uses, as input, visible keypoints of individual animals provided by a fully-convolutional detector. Individual animals are also equipped with ear tags that are used by a classification network to assign unique identification to instances. The fixed cardinality of the targets is leveraged to create a continuous set of tracks and the forward-backward algorithm is used to assign ear-tag identification probabilities to each detected instance. Tracking achieves real-time performance on consumer-grade hardware, in part because it does not rely on complex, costly, graph-based optimizations. A publicly available, human-annotated dataset is introduced to evaluate tracking performance. This dataset contains 15 half-hour long videos of pigs with various ages/sizes, facility environments, and activity levels. Results demonstrate that the proposed method achieves an average precision and recall greater than 95% across the entire dataset. Analysis of the error events reveals environmental conditions and social interactions that are most likely to cause errors in real-world deployments.

## 1. Introduction

It is necessary to observe animals on an individual level in order to assess their health and wellbeing and ensure efficient production. One of the most significant challenges to industry is its reliance upon subjective human observation for assessment, which can be as low as only a few seconds per animal each day [1]. This challenge is enhanced when symptoms are subtle and the mere presence of humans encourages animals to alter or mask individual symptoms to disguise signs of illness/injury [2,3,4]. Despite the fact that researchers have been able to identify links between health and behavior [5,6,7], the limitations of human observation make it difficult to achieve a timely diagnosis of compromised animals and intervene on their behalf [8].

A technological solution that augments and expands beyond the limitations of human observation could address many of these challenges. To this end, a goal within the precision livestock farming movement is to invent new technologies for continuous recording of animal activities and devise ways of using that data to predict outcomes for individual animals [9,10,11]. Early attempts to achieve this goal often involved attaching electronic devices to individual animals. This includes both active devices like ultra-wide band (UWB), inertial measurement units, and GPS [12,13,14,15,16,17,18,19,20] as well as passive electronic devices like radio-frequency identification (RFID)  [21,22,23,24]. As a result that they must be physically attached to the animals, these are invasive devices that impact animal welfare. Furthermore, due to financial constraints and concerns about durability and hardware management [25], modern approaches to precision livestock farming are trending toward non-invasive, vision-based solutions [26,27,28,29].

The shift toward vision-based solutions is largely due to advancements over the past decade in deep learning [30]. The rediscovery of convolutional neural networks [31], along with software for efficiently training the networks [32,33], has made it possible to create vision systems that acquire a highly sophisticated understanding of images and video by simply being trained on large human-annotated datasets [34,35]. Numerous datasets exist for common tasks like image classification, semantic segmentation, person pose estimation, and street-scene understanding [36,37,38,39,40]. However, for less common applications like livestock tracking, publicly available datasets are scarce and many researchers resort to demonstrating their methods on small, private datasets. This practice makes it difficult to evaluate the performance of new methods and chart progress in the field.

**Figure 1:**
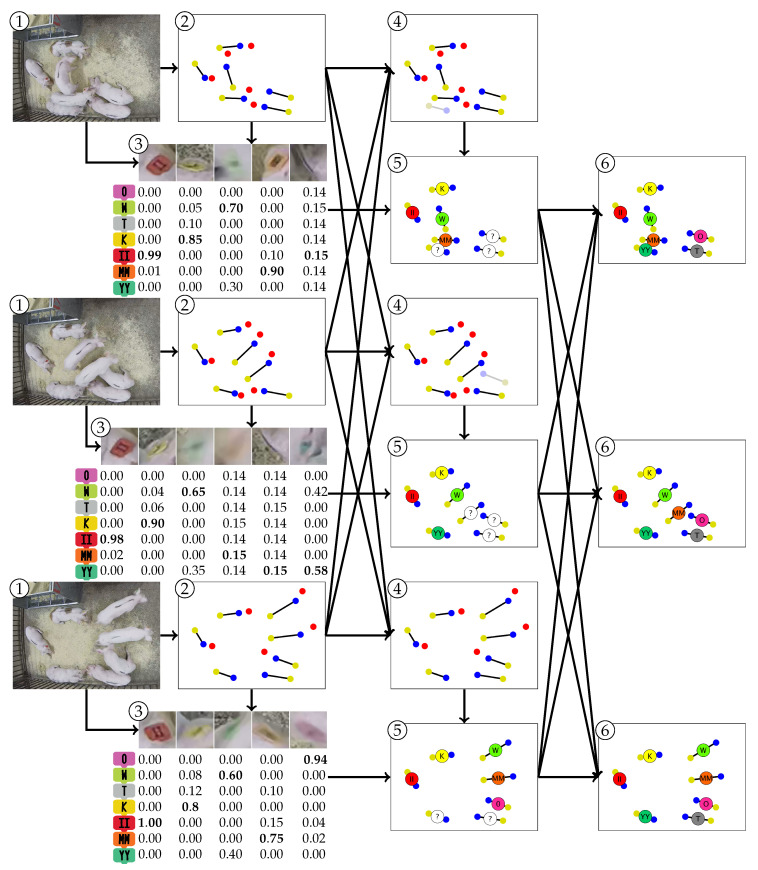
Three consecutive frames illustrating the six stages of processing. The input image (1) are processed using the detection method to find the locations of pigs, as defined by their matched shoulder-tail coordinates (2). Detected ear locations (2) are used to extract small image crops that can be used for ear tag classification (3). Here, there are seven possible ear tag IDs and each cropped image is mapped to a probability vector (3). The original detections (2) across all frames are used to interpolate missing detections (4). The ear tag probability vectors are mapped to all detections to initialize the ID probabilities prior to inference (5). Finally, ear tag ID probabilities are shared across frames using forward-backward MAP estimation to derive consistent IDs.

A method is proposed here for long-term tracking of multiple group-housed livestock using computer vision. Figure 1 illustrates the processing stages of the method on three consecutive frames of video. The method begins with a deep, fully-convolutional network that processes every frame of video to detect individual animals as collection of anatomical features (i.e., keypoints, shown in stage 2 in Figure 1). Here, shoulder-tail combinations are used to identify the target locations and orientations in each frame and ears are used to determine the ID of each target. The last stage of processing that happens on a per-frame basis is ear tag classification (stage 3 in Figure 1), where small image crops around each detected ear location are converted to probability vectors using a classification network. To handle detections that are missed due to occlusions and/or challenging presentations, an efficient interpolation method is used that relies on the fact that the number of targets is fixed and known to the tracking algorithm. Interpolation results are illustrated by the addition of faded detections in stage 4 of Figure 1, where the position of the faded detections is inferred from other frames in the sequence. Finally, the ear tag probabilities are used to initialize the likelihoods at each interpolated location (stage 5 in Figure 1) and maximum a posteriori (MAP) estimation is used to share probabilities between frames (stage 6 in Figure 1).

Essentially, the final stage of processing is used to merge tracking-by-detection results with target ID classification using the assumption that target locations are consistent between frames. For example, in Figure 1 the pink O ear tag is only seen in one frame, but its location in that frame is the nearly identical to its location in all other frames, so the other locations inherit the pink O classification. The gray T ear tag is never seen or classified, however, through the process of elimination the pig in the lower right of the frames has the highest probability of belonging to this class.

The method is evaluated on a new, publicly available, human-annotated dataset that contains fifteen 30-min videos. The collection of videos depicts different animal ages/sizes, variations in housing facilities, basic activity levels, and lighting scenarios. It was designed to challenge tracking methods in a wide range of situations. This includes situations with challenging visibility conditions when, for example, young pigs tend to congregate (pile up together) at night as a means to share body heat. On the opposite side of the spectrum, it includes challenging tracking situations where older pigs frantically chase each other around the pen. It also includes relatively easy situations when, for example, large pigs calmly move throughout the pen space during the day. Videos were captured at five frames per second and a total of  135,000 frames were annotated and used in the evaluation. Overall, the proposed method achieves tracking precision and recall above 52% in the most challenging situations and, in more than half of tested cases, exceeds 98% in both precision and recall. Key contributions of this work include (1) complimentary methods for detection and classification using convolutional neural networks, (2) a probabilistic framework for merging classification likelihoods to detections, and (3) a publicly available dataset for training and evaluating long-term tracking methods under a variety of challenging situations.

This paper continues by presenting related work in animal tracking in Section 2. The proposed method is then introduced in Section 3. Section 4 describes a new human-annotated dataset and benchmark used to evaluate vision-based tracking methods. The results of the proposed method on the dataset are also discussed in Section 5. Finally, Section 6 provides concluding remarks and directions for future research on this topic.

## 2. Background

Tracking group-housed livestock is a challenging task that necessitates novel solutions. Existing methods for tracking pedestrians provide a wide range of useful techniques, however, they are designed around a set of assumptions that do not generally hold for group-housed livestock. They often assume that first or second order movement models can be used to separate targets as they move through the scene [41,42]. While this may be true for short time segments, movement models are incapable of overcoming the inevitability of swaps and lost targets due to occlusions. To recover from these inevitable failure cases, existing methods are trending toward deep feature-based target re-identification [43,44]. However, the ability to re-identify a target based upon unique features breaks down when the targets are homogeneous (lacking discernible physiological differences), as is often the case for livestock populations.

To address these challenges, researchers have taken a variety of different approaches. The method introduced by Nasirahmadi et al. [45] characterizes group behaviors using shape fitting techniques [46] customized to their targets. Although variations in the environment and presentation of the animals were limited, they were able to demonstrate accurate multi-target detection of group-housed pigs. One of the first attempts at using supervised learning to detect and track group-housed pigs was introduced by Nilsson et al. [47]. Their results, while promising, were limited to ideal viewing conditions and the method was not designed to handle occlusions.

With the introduction of the Microsoft Kinect depth camera [48], researchers began leveraging depth camera capabilities for animal tracking [49,50,51,52,53,54,55,56,57,58,59]. Not only do depth cameras make it relatively easy to separate foreground objects from a static background, but they also make it possible to track objects using known properties of their three-dimensional shapes. One example was introduced by Ju et al. [27], where targets were first detected using the YOLO network [60] and then a separate stage of post-processing was used to separate objects with shared bounding box areas. This method demonstrated a high level of accuracy (92%), but it was limited to three group-housed pigs. An alternative approach by Mittek et al. [61] used iterative ellipsoid-fitting to track target locations and orientations. The method provides an average of 20 min of continuous tracking without errors, however, the lack of an accompanying detection method meant that each pig’s location needed manual initialization prior to operation. Furthermore, the method does not include a way to recover from error events or re-identify targets in the event that they are swapped or lost.

Arguably the most important contributing factor to a tracking method’s success is the performance of its detector [62]. Fortunately, detection accuracy has improved markedly over the past ten years due to methods like R-CNN [63], YOLO [60], and Mask R-CNN [35]. Furthermore, methods that detect objects as collections of joined parts, such as OpenPose [64] and PersonLab [65], make it possible to infer the location and pose of targets. This has significant implications for animal tracking, as it makes it possible to more reliably associate detections across frames of video and it provides more details regarding target activities and social interactions. One of the first attempts to detect animals as a collection of parts was introduced by Ardö et al. [26]. They trained and applied a neural network to detect keypoints of cattle that were visible from a top-down view. Results demonstrated that the method was capable of 95% accuracy in the trained environment, but dropped to 55% when applied in new environments not seen during training.

The method introduced by Psota et al. [28] provides a method for detecting and associating the body part locations of pigs using a fully-convolutional neural network. By representing targets as a collection of body parts, their method can infer more detailed activities and social interactions than would be possible with bounding-box approaches. They also released a publicly available dataset with 2000 annotated images of 24,842 individually pigs from 17 different locations. Results demonstrated that the method could achieve a precision of 0.99 and a recall of 0.96 when the network was trained on the environment. This performance dropped when applied to new environments, demonstrating the importance of fine-tuning with new data.

Zhang et al. [29] proposed a method to detect pigs and associate them across frames using a combination of trainable methods. Detection is based on the architecture of the Single-Shot Detector (SSD) [66] and it is used to identify pigs via a location near the middle of their backs, which they refer to as “tag-boxes.” To associate detections between frames, they apply a trainable correlation filter to the tag-box regions to track pigs as a single feature point in the images. Results are presented on a dataset containing five videos averaging 39 s in duration. The conditions in the videos are varied, however, they consistently depict nine large (finisher) pigs. While the results are promising, the method does not include a method for absolute identification. Therefore, it can not be expected to achieve reliable long-term tracking.

This paper introduces a long-term tracking strategy that leverages the high-precision detection outputs provided by [28]. Despite the sophistication of modern motion modeling and target association methods, long-term tracking is bound to fail at some point. This can be due to the complex movements and interactions between targets, camera obstructions, or power outages. Recovery from these inevitable tracking failures is a daunting challenge when the targets are as visually indistinguishable as group-housed pigs. To address this challenge, the proposed method augments the appearance of each pig with visually distinguishable ear tags. The ear tags, while not always visible, provide intermittent opportunities to recover from tracking failures, i.e., when target IDs are swapped. A MAP estimation framework is derived to problematically merge the outputs of tracking-by-detection with ID information provided by ear tag observations.

## 3. Method

The proposed multi-object tracking method is designed for animals living in fixed group-house environments. While pigs were used in this study to develop the techniques and evaluate the performance, the methodology applies to a variety of targets that satisfy the following assumptions.

1. Video footage is obtained from a static camera mounted above the environment of interest.
2. The field of view of the camera encompasses the entire living space.
3. The number of targets remains constant and each is equipped with a unique visual marker.

**Figure 2:**
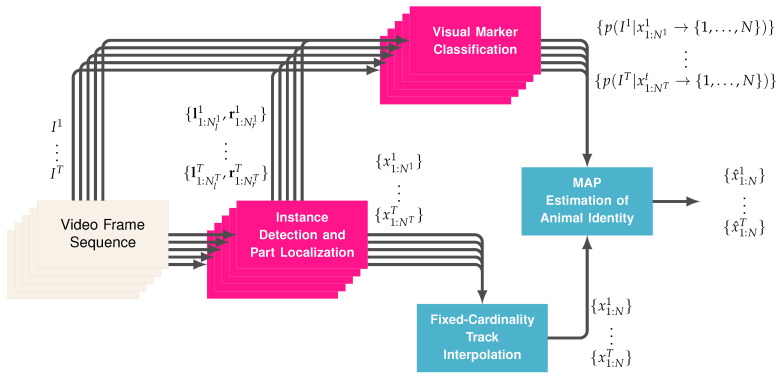
Flow diagram of the proposed method. The input consists of a sequence of consecutive frames $\left\{I^{1},\ldots ,I^{T}\right\}$. Information extracted from the frames is used to detect and track individual targets as well as classify their identities using visual marker classification. Finally, the last stage merges tracks with identify likelihoods to achieve maximum a posteriori (MAP) estimation of the location of each target in the frames, denoted $\left\{\left\{{\hat{x}}_{1:N}^{1}\right\},\ldots ,\left\{{\hat{x}}_{1:N}^{T}\right\}\right\}$.

The processing steps of the proposed method are illustrated in Figure 2 and each is referred to as a module. This section begins with an overview of each module and an introduction to the notation used throughout this paper. The details of the modules are then provided in Section 3.1–Section 3.4.

The method begins with a video represented by the set of images $I^{1:T}=\left\{I^{1},\ldots ,I^{T}\right\}$, where *T* is the number of consecutive images in the video sequence. First, the images are processed by the Instance Detection and Part Localization module to detect targets and extract the image coordinates of each instance. Specifically, for the $t^{\mathrm{th}}$ frame, the set of $N^{t}$ instances detected by the module are denoted $x_{1:N^{t}}^{t}=\left\{x_{1}^{t},\ldots ,x_{N^{t}}^{t}\right\}$. Note that the pig index *n* for $x_{n}^{t}$ does not correspond with the true identity of the pig. Rather, this is simply an index indicating the order in which it was detected in frame *t* and, at this stage, no correspondence is assumed between $x_{n}^{t}$ and $x_{n}^{t+1}$. In this work, the shoulder and tail locations define each instance, so $x_{n}^{t}=\left\{s_{n}^{t},t_{n}^{t}\right\}$, where $s_{n}^{t}$ is the two-dimensional image coordinate of the shoulder for instance *n* in frame *t* and $t_{n}^{t}$ is the corresponding coordinates of the tail.

The Instance Detection and Part Localization module also detects the locations of all visual markers. In this work, the visual markers correspond to physical ear tags in the left and/or right ears. In frame *t*, the collection of two-dimensional image coordinates of left ears is defined as $l_{1:N_{l}^{t}}^{t}=\left\{l_{1}^{t},\ldots ,l_{N_{l}^{t}}^{t}\right\}$ and the collection of right ear tag coordinates is defined as $r_{1:N_{r}^{t}}^{t}=\left\{r_{1}^{t},\ldots ,r_{N_{r}^{t}}^{t}\right\}$. Note that the estimated number of visual markers $N_{l}^{t}$ and $N_{r}^{t}$ can be greater or less than the number of detected instances $N^{t}$. For each detected visual marker location, a crop is taken from the original image around that marker’s location. This cropped image is then passed through the *Visual Marker Classification* module to predict the class membership of the visual marker and associate that prediction with the set of instances. The output of this module is a likelihood $p(I_{t}|x_{n}^{t}\to \left\{1,\ldots ,N\right\})$ that image $I_{t}$ was observed given that the detected instance $x_{n}^{t}$ has an ID of 1,2,…,N.

Ideally, the number of detected instances $N^{t}$ for any given frame *t* will be equal to the known number of targets, *N*. However, the detector will miss some instances (false negatives) and also detect instances in incorrect locations (false positives). The Fixed-Cardinality Track Interpolation module processes the output of the detector and produces a fixed number of targets in each frame. It begins by limiting the number of detection in each frame by removing the least confident detections so that $N^{t}\leq N\;\forall \;t=1,\ldots ,T$. Then, the module associates detections between frames into continuous tracks and interpolates target locations when detections are missing. The result is *N* continuous tracks that span the entire video sequence.

Finally, the output of the Visual Marker Classification module is combined with the continuous tracking output of the Fixed-Cardinality Track Interpolation module to estimate the most likely IDs associated with each detection. This process takes place in the MAP Estimation of Animal Identity module. The output of the module is an ordered set of detections $\left\{{\hat{x}}_{1,1:N}\right\},\ldots ,\left\{{\hat{x}}_{1:N}^{t}\right\}=\left\{{\hat{x}}_{1,1},\ldots ,{\hat{x}}_{1,N}\right\},\ldots ,\left\{{\hat{x}}_{1}^{t},\ldots ,{\hat{x}}_{N}^{t}\right\}$, where ${\hat{x}}_{n}^{t}$ indicates the location of target *n* in the $t^{\mathrm{th}}$ frame.

### 3.1. Instance Detection and Part Localization

Tracking-by-detection methods begin with a per-frame detector that finds the location of individual instances. In this work, the detection method produces a set of instance locations $\left\{x_{1:N^{t}}^{t}\right\}=\left\{x_{1}^{t},\ldots ,x_{N^{t}}^{t}\right\}$ for each frame t=1,…,N. Furthermore, each instance is defined by its two-dimensional, image-space, pairwise shoulder, and tail location, which can be represented by $x_{n}^{t}=\left\{s_{n}^{t},t_{n}^{t}\right\}$. The detection method also detects the locations of visual markers in the image space. In this work, these locations correspond to the pixel coordinates of the left and right ears of the pigs, denoted $l_{1:N_{l}^{t}}^{t}=\left\{l_{1}^{t},\ldots ,l_{N_{l}^{t}}^{t}\right\}$ and $r_{1:N_{r}^{t}}^{t}=\left\{r_{1}^{t},\ldots ,r_{N_{r}^{t}}^{t}\right\}$, respectively.

**Figure 3:**
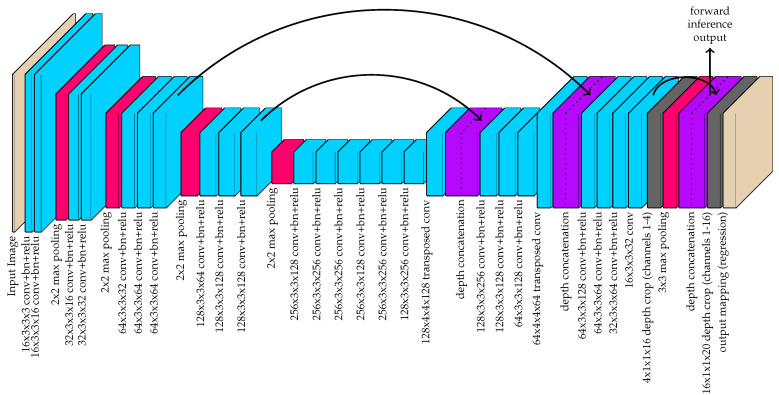
The hourglass-shaped network used by the proposed method to convert images to 16-channel image-space instance detection maps. The black arrows indicate a copy for depth concatenation. The gray depth crop layers indicate untrainable convolutional layers that isolates certain channels of the input. While the final output layer is used for training with MSE regression, the output of the 3rd depth concatenation is used for forward inference. By including the 3 × 3 max pooling output of the channels 1–4 along with their original outputs, this allows for fast peak detection in post-processing.

The proposed detection method is largely based on the method presented in [28], and the network architecture is illustrated in Figure 3. Instead of using the original network with maxpooling and maxunpooling layers, the network used in this work adopts a more efficient U-net architecture [67]. This architecture is characterized by the use of depth concatenations following transposed convolutions for upsampling. The depth concatenations serve two key purposes. First, this allows for accelerated training because there are more direct paths from the network output to earlier layers in the network. This advantage was first demonstrated by the ResNet [34] architecture and, subsequently, the DenseNet [68] architecture. The second function of the depth concatenations is to allow the network to produce fine details in the output feature space. Early maxpooling layers remove spatial details and make it difficult for transposed convolutions to produce detailed outputs at higher resolutions. Thus, by concatenating the network output prior to maxpooling after each transposed convolution, the network has access to higher resolution details.

It is worth noting that the DeepLabV3+ [69] architecture was also considered for this application. DeepLabV3+ is characterized by the use of atrous convolutions to preserve the feature-space resolutions of networks like ResNet [34] that natively downsample by a factor of 64 from, for example, a 224 × 224 input to a 7 × 7 feature space. Instead of drastically downsampling the network, the atrous convolutions expand the reach of convolutions, making it possible to preserve the receptive field while maintaining spatial precision with a larger feature space. Furthermore, the DeepLabV3+ network finishes by processing the feature space with a set of narrow and wide atrous convolutions so that the network is able to reuse features from multiple scales instead of having to train feature extraction differently for big and small objects.

Despite the popularity of the DeepLabV3+ network architecture for semantic segmentation tasks, it was empirically deemed to be unsuitable for this application. This was due to its inability to recover fine spatial details in the output feature space. It is likely that the strength of this architecture—its ability to detect objects regardless of scale—was not critical to this application. While the targets do vary in size, the consistent camera setup and relatively homogeneous presentation of the targets made this application much different than such things as segmenting images from the COCO dataset. In addition, fine detail is critical for the animal tracking application, but it is not critical to achieve high scores on semantic segmentation benchmarks where 50% intersection over union (IoU) is sufficient for detection.

There are three major changes to the architecture presented in [28] that make this network more efficient. First, as discussed earlier, maxunpooling layers were removed and replaced with transposed convolutions. Maxunpooling operations are generally slower because they require the network to pass indices that vary from one image to another. The second major change is that the output is left at a 4× down-sampled resolution instead of upsampling all the way back up to the original resolution. The objects/parts being detected are expected to be strictly larger than a 4 × 4 window in the input image resolution and sub-pixel interpolation is used to detect the real-valued locations within the feature space. Thus, this lower resolution output has sufficient spatial detail and it removes the burden of computing regional maximums over large image spaces. Finally, the third major change is that the regional maximum values for the channels corresponding to body part locations are calculated within the network structure by a 3 × 3 maxpooling layer. This regional maximum computation happens on the GPU during forward inference, and it adds a negligible increase to the time required by the GPU to process each image. Regional maximums are used to find local maximum responses indicating the pixel locations of target body parts. By performing maxpooling in-network and concatenating this output with the body part mapping prior to maxpooling, region maximums can be quickly found with simple pixel-wise “is equal” comparisons in post-processing.

### 3.2. Fixed-Cardinality Track Interpolation

After detecting instances using the method described in Section 3.1, the sequence of detected target locations $\left\{x_{1:N^{1}}^{1},\ldots ,x_{1:N^{T}}^{t}\right\}$ is used to construct continuous tracks for exactly *N* targets. The proposed tracking method begins by removing high-cost detections whenever $N^{t}>N$ for all t=1,…,T. Here, cost is defined for each instance using

(1) $$C\left(x_{n}^{t}\right)=\frac{{|\left(t\to s\right)}_{n}^{t}-s_{n}^{t}{|+|\left(s\to t\right)}_{n}^{t}-t_{n}^{t}|}{2\times |s_{n}^{t}-t_{n}^{t}|\times \left(\mathrm{score}\left(s_{n}^{t}\right)+\mathrm{score}\left(t_{n}^{t}\right)\right)},$$

where $s_{n}^{t}$ and $t_{n}^{t}$ are the two-dimensional shoulder and tail coordinates that define the location of the instance. Furthermore, ${\left(t\to s\right)}_{n}^{t}$ is the estimated shoulder coordinates taken from the tail coordinate $t_{n}^{t}$, and ${\left(s\to t\right)}_{n}^{t}$ is the estimated tail coordinates taken from the shoulder coordinate $s_{n}^{t}$. These estimates and their use in detecting instances are discussed in detail in [28]. The metrics $\mathrm{score}(s_{n}^{t})$ and $\mathrm{score}(t_{n}^{t})$ are the outputs in the shoulder and tail detection channels of the network output. When the shoulder and tail location estimates are perfect, i.e., ${\left(t\to s\right)}_{n}^{t}=s_{n}^{t}$ and ${\left(s\to t\right)}_{n}^{t}=t_{n}^{t}$, the cost $C(x_{n}^{t})=0$. In addition, the cost of an instance increases as the score of the shoulder and tail detection decrease. It is worth noting that the minimum values of $\mathrm{score}(s_{n}^{t})$ and $\mathrm{score}(t_{n}^{t})$ are lower bounded to 0.25 so the most that these terms can increase the cost is by a factor of 2. When they are below 0.25, these parts are not detected and cannot contribute to an instance. In contrast, when they are both equal to one, the cost is decreased by a factor of 2.

**Figure 4:**
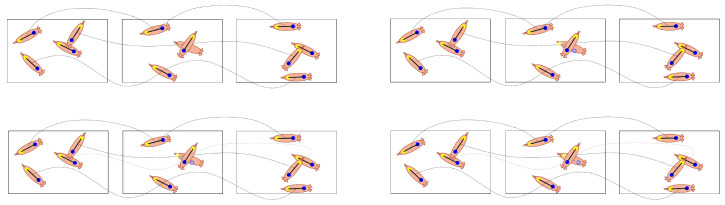
The figures illustrate a situation with four pigs in three consecutive frames. In the first stage (**top-left**), three of the four pigs are tracked and one is missed in the middle frame due to partial occlusion. As a result that there is an unassigned pig from the previous frame, that pig’s location is duplicated and marked as a duplicate (**top-right**). With a consistent number targets per frame, the Hungarian algorithms is used to join targets between frames (**bottom-left**). Finally, the locations of the duplicates are interpolated smoothly between detections (**bottom-right**).

Once the detections per frame are limited to $N^{t}\leq N$ for all t=1,…,T, a set of *N* continuous tracks can be approximated using Algorithm 1. Figure 4 illustrates a simple example of the algorithm’s stages for four targets in three consecutive frames. The first step in the process is to scan the frames from 1 to *T* and duplicate detections from the previous frame any time they are not assigned to the next frame via the Hungarian algorithm. In the second stage, the frames are processed in the reverse order and duplicates are again created for missed assignments. All links between detections created via the Hungarian algorithm are stored in memory and, after the second stage, there will be *N* continuous tracks across all *T* frames. When a duplicate is created in either the first or second stage, it is marked as a duplicate. Finally, the locations of duplicates are interpolated by finding the nearest detections looking forward and backward in time and creating the interpolated location by weighing the detected locations by their distance in time from the duplicate. The equation for the interpolated position is given in Algorithm 1 and an example of how this might change the position of the duplicate is illustrated in the bottom two sequences of Figure 4.

**Algorithm 1:** Fixed-Cardinality Track Interpolation.

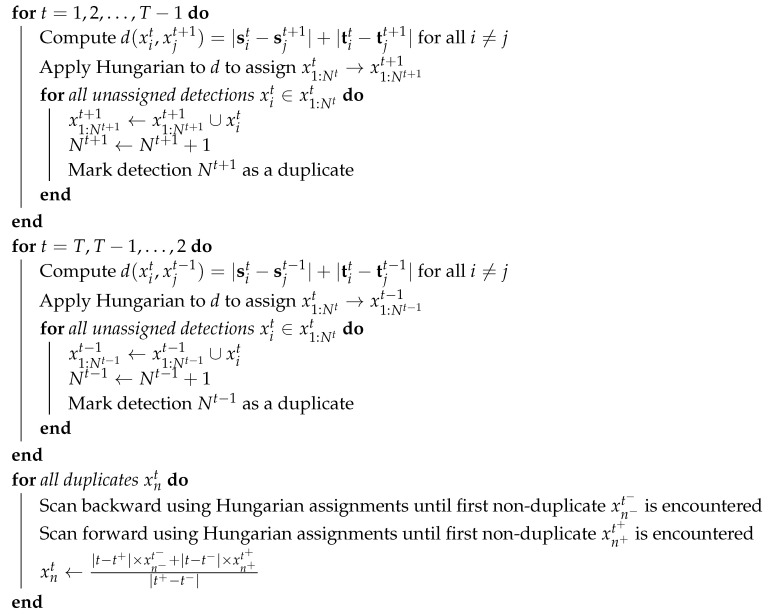



### 3.3. Visual Marker Classification

**Figure 5:**
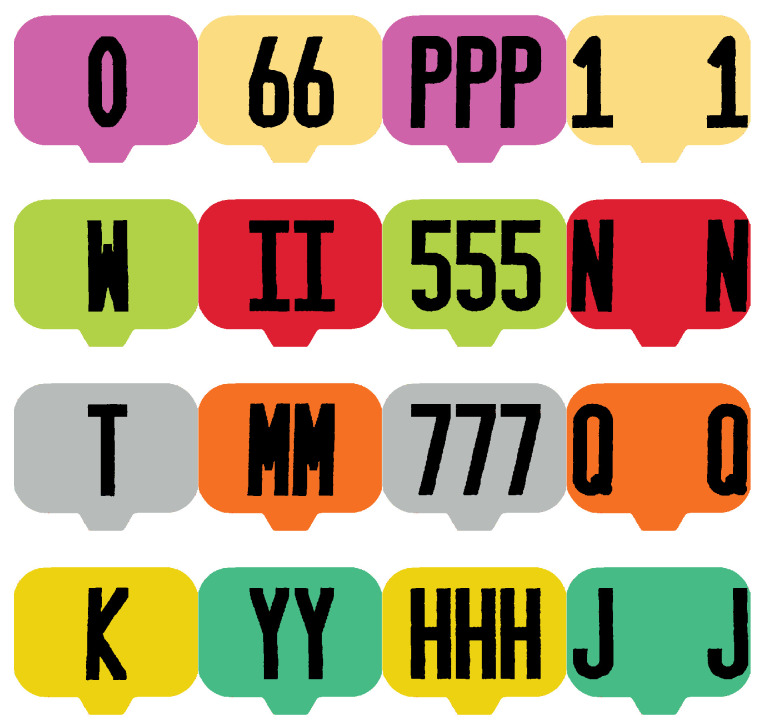
The 16 unique ear tags used in this work to identify individual pigs. The tags use alphanumeric characters printed on Destron Fearing ${}^{\mathrm{TM}}$ Hogmax ear tags.

In applications where unique visual identification of animals is important, it is common for livestock to be issued permanent ear tags. Serial numbers are common, however, they are not ideal for visual identification. Therefore, a different set of tags was designed and used in this work. The set of 16 tags, illustrated in Figure 5, includes a variety of different color/alphanumeric character combinations. The specific combination chosen here was intended to be easily recognizable for people, even in difficult viewing conditions.

**Figure 6:**
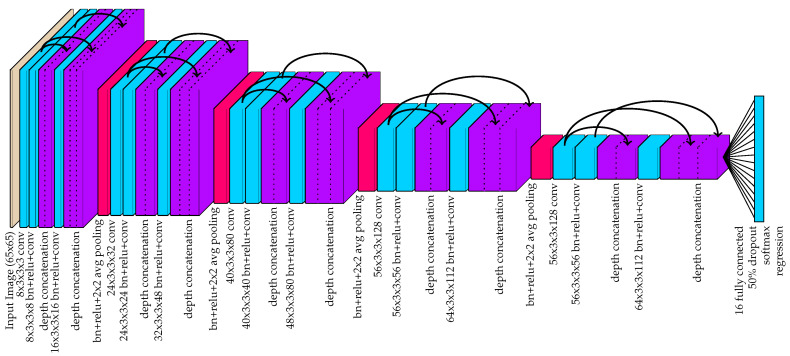
The deep neural network used to classify tags from 64 × 64 crops at the ear locations.

For the proposed tracking system, the tags serve as an absolute way to identify each animal and recover from tracking errors. When an ear is located in the image, that section of the image centered at the ear is cropped to a 65 × 65 image. The cropped image is then processed by the convolutional neural network shown in Figure 6 to provide a likelihood that the observed ear is equipped with one of the known tags. The network was designed using the DenseNet architecture [68] (with k=8).

At each time step *t* an observation $I^{t}$ is made regarding the specific identity of each left or right ear location, denoted $r_{i}^{t}$ or $l_{i}^{t}$, respectively. The ear location will be denoted $e_{i}^{t}$ to simplify notation, and any operation that applies to $e_{i}^{t}$ applies to both $r_{i}^{t}$ and $l_{i}^{t}$. In this case, the observation is confined to a 65 × 65 window around the animal’s ear. The trained network uses this observation to derive the probability $p(e_{i}^{t}\to \left\{1,\ldots ,N\right\}|I^{t})$ of ear tag $e_{i}^{t}$ having identity {1,…,N}, given an observation $I^{t}$.

Target instances are defined by pairs of shoulder and tail locations. The network provides association vectors to predict the locations of shoulders from both the right and left ear. Thus, instead of making hard decisions regarding which ear belongs to which instance, the association vectors are used to evaluate the probability that an ear tag belongs to an instance. Specifically, the average back-and-forth distance between ears and shoulders is found using

(2) $$d\left(s_{i}^{t},e_{j}^{t}\right)=\frac{{|\left(e\to s\right)}_{j}^{t}-s_{i}^{t}{|+|\left(s\to e\right)}_{i}^{t}-e_{j}^{t}|}{2\times |s_{i}^{t}-e_{j}^{t}|}.$$

As this distance increases, the probability that the ear is linked to the shoulder is decreased with a decaying exponential given by

(3) $$p\left(s_{i}^{t}\to e_{j}^{t}\right)=\max\left(10^{-6},e^{-10\times d(s_{i}^{t},e_{j}^{t})}\right),$$

where a lower bound of $10^{-6}$ prevents network over-confidence from creating instability.

Finally, the probability $p(x_{i}^{t}\to \left\{1,\ldots ,N\right\}|I^{t})$ of assigning a specific identity to an instance is initialized with a uniform probability of 1/N and, for each tag and each detected instance, the probability is modified using a weighted summation of the network output and the uniform probability. This calculation is given by

(4) $$p\left(x_{i}^{t}\to n|I^{t}\right)=\frac{1}{N}\prod_{j=1}^{N_{e}^{t}}\left(p\left(s_{i}^{t}\to e_{j}^{t}\right)p\left(e_{j}^{t}\to n|I^{t}\right)+\left(1-p\left(s_{i}^{t}\to e_{j}^{t}\right)\right)\frac{1}{N}\right).$$

In the extremes of $p(s_{i}^{t}\to e_{j}^{t})$, this results in $p\left(x_{i}^{t}\to n|I^{t}\right)\approx \frac{1}{N}$ when none of the tag locations are strongly linked to the instance location and it results in $p\left(x_{i}^{t}\to n|I^{t}\right)\approx p\left(e_{i}^{t}\to n|I^{t}\right)$ when ear tag $e_{j}^{t}$ is a highly confident match to instance location $x_{i}^{t}$. It should also be noted that $p\left(x_{i}^{t}\to n|I^{t}\right)\propto p\left(I^{t}|x_{i}^{t}\to n\right)$ when all tags are equally likely to be observed and, for the purposes of optimization, the probability of the observation does not affect probability maximization.

### 3.4. Maximum A-Posteriori (MAP) Estimation of Animal Identity

In livestock tracking applications with frame rates exceeding 4 fps, targets move very little between frames. Therefore, a “stay put” motion model is adopted here. Let $p(x_{i}^{t}|x_{j}^{t-1})$ be the probability of transitioning to state $x_{i}^{t}$ given that the tracked target was previously in state $x_{j}^{t-1}$, and let the distance between $x_{i}^{t}$ and $x_{j}^{t-1}$ be defined as

(5) $$\delta \left(x_{i}^{t},x_{j}^{t-1}\right)=\sqrt{|s_{i}^{t}-s_{j}^{t}{|}^{2}+{\left|t_{i}^{t}-t_{j}^{t}\right|}^{2}}.$$

**Figure 7:**
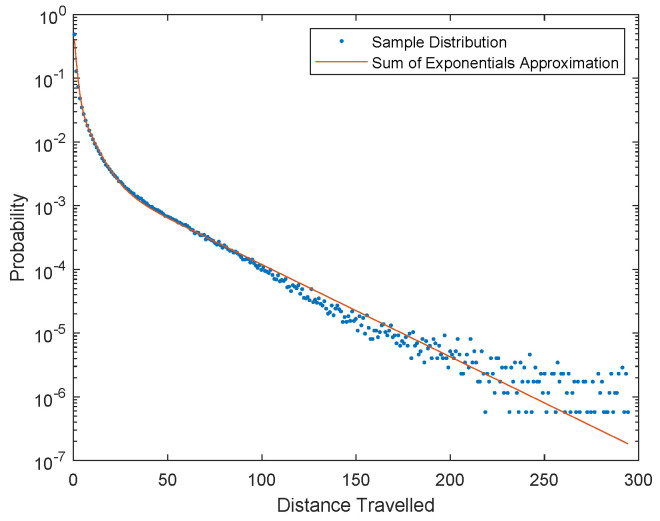
The distribution of Euclidean distances between targets in neighboring frames (blue dots) is approximated using a weighted sum of exponential distributions (solid orange line).

Using a labeled dataset, described in detail in Section 5, a set of 1.73 million samples was collected and its distribution is given by the blue dots in Figure 7. This distribution can not be closely approximated by a single exponential distribution. Instead, it requires a weighted sum of three exponential distributions to achieve the approximation illustrated by the orange line in Figure 7. The equation for the approximate distribution is

(6) $$p\left(x_{i}^{t}|x_{j}^{t-1}\right)\approx 0.6\times \frac{9}{10}e^{-9\delta (x_{i}^{t},x_{j}^{t-1})/10}+0.3\times \frac{1}{6}e^{-\delta (x_{i}^{t},x_{j}^{t-1})/6}+0.1\times \frac{1}{30}e^{-\delta (x_{i}^{t},x_{j}^{t-1})/30}.$$

Equation (4) provides the likelihood of the observation given a specific identity for the target and Equation (6) provides the probability of a target transitioning between frames from one location to another. Together, these two probabilities make it possible to calculate the Maximum A-Posteriori (MAP) estimate of each target’s identity.

The proposed method aims to evaluate the probability that target *n* exists in state $x_{i}^{t}$ given the entire sequence of observations $\left\{I^{1},\ldots ,I^{T}\right\}$. This probability, previously denoted $p(x_{i}^{t}\to n|I^{t})$, will now be shortened to $p(x_{i}^{t}|I^{t})$ to simplify notation. As a consequence, it is assumed that the following operations are performed separately for all n=1,…,N. If we assume conditional independence between past and future observations given the current state, the probability can be represented by

(7) $$p\left(x_{i}^{t}|I^{1:T}\right)=p\left(x_{i}^{t}|I^{1:t},I^{t+1:T}\right)=\frac{p(x_{i}^{t},I^{1:t},I^{t+1:T})}{p(I^{1:t},I^{t+1:T})}=\frac{p\left(I^{1:t}|x_{i}^{t}\right)p\left(x_{i}^{t}|I^{t+1:T}\right)p\left(I^{t+1:T}\right)}{p\left(I^{1:t}\right)p\left(I^{t+1:T}\right)},$$

where $I^{a:b}=\left\{I^{a},\ldots ,I^{b}\right\}$ is used to simplify notation. The probability of the observations themselves do not affect maximization, thus the expression can be further reduced to

(8) $$p\left(x_{i}^{t}|I^{1:N}\right)\propto p\left(I^{1:t}|x_{i}^{t}\right)p\left(x_{i}^{t}|I^{t+1:T}\right).$$

This set of posterior marginals can be found using the forward-backward algorithm, which operates by sequentially computing the forward probabilities $\alpha^{t}\left(x_{i}^{t}\right)=p\left(I^{1:t}|x_{i}^{t}\right)$ and backward probabilities $\beta^{t}\left(x_{i}^{t}\right)=p\left(x_{i}^{t}|I^{t+1:T}\right)$ at each time step t=1,…,T. The update equation for the forward probabilities is given by

(9) $$\alpha^{t}\left(x_{i}^{t}\right)=p\left(I^{t}|x_{i}^{t}\right)\sum_{j=1}^{N}\alpha^{t-1}\left(x_{j}^{t-1}\right)p\left(x_{i}^{t}|x_{j}^{t-1}\right),$$

where $\alpha_{1}\left(x_{i}^{1}\right)=p\left(I^{1}|x_{i}^{1}\right)$. For backward probabilities, the sequential update equation is

(10) $$\beta^{t}\left(x_{i}^{t}\right)=\sum_{j=1}^{N}\beta^{t+1}\left(x_{j}^{t+1}\right)p\left(x_{i}^{t}|x_{j}^{t+1}\right)p\left(I^{t+1}|x_{j}^{t+1}\right),$$

where $\beta_{1}\left(x_{i}^{1}\right)=1\;\forall \;k=1,\ldots ,N$. Finally, the posterior marginal probability can be computed at each time step as

(11) $$p\left(x_{i}^{t}|I^{1:N}\right)\propto \alpha^{t}\left(x_{i}^{t}\right)\beta^{t}\left(x_{i}^{t}\right).$$

In theory, the standard form of the forward-backward algorithm is suitable for evaluating and comparing the probabilities of target memberships. In practice, however, when implemented in software with floating point precision variables, underflow becomes an unavoidable problem. Essentially, the magnitudes of probabilities become so low that they reach the lower limit of the variable type and are either forced to zero or set to a fixed lower bound. In either case, the value of the probabilities is no longer accurate, creating instability in the system.

To avoid underflow, the forward-backward algorithm can be implemented using the log-sum-exp method [70]. This approach operates by adding the logarithms of the probabilities instead of multiplying them, creating a much wider dynamic range. However, the fact that the original expressions for the forward and backward term include summations makes it necessary to add an additional exponent and logarithm. The expression for the logarithm of the forward term becomes

(12) $$\log\left(\alpha^{t}\left(x_{i}^{t}\right)\right)=\log\left(p\left(I^{t}|x_{i}^{t}\right)\right)+\log\left(\sum_{j=1}^{N}\exp\left(\overset{a_{x^{t-1}}}{\overset{︷}{\log(\alpha^{t-1}\left(x_{j}^{t-1}\right)+\log\left(p\left(x_{i}^{t}|x_{j}^{t-1}\right)\right)}}\right)\right).$$

In this expression, there remains a significant risk of underflow when the values of $a_{x^{t-1}}$ become large magnitude negative numbers. For this reason, the value $a_{\max}=\max_{x^{t-1}}a_{x^{t-1}}$ is computed and subtracted from each term within the summation. The revised expression

(13) $$\log\left(\alpha^{t}\left(x_{i}^{t}\right)\right)=\log\left(p\left(I^{t}|x_{i}^{t}\right)\right)+\log\left(\sum_{j=1}^{N}\exp\left(\log\left(\alpha^{t-1}\left(x_{j}^{t-1}\right)\right)+\log\left(p\left(x_{i}^{t}|x_{j}^{t-1}\right)\right)-a_{\max}\right)\right)+a_{\max}$$

sets the largest value of arguments within the exponent to zero and then adds back the value of $a_{\max}$ outside of the summation. The following two expressions for the logarithm of the backward term perform an equivalent set of tricks to avoid underflow.

(14) $$\log\left(\beta^{t}\left(x_{i}^{t}\right)\right)=\log\left(\sum_{j=1}^{N}\exp\left(\overset{b_{x_{j}^{t+1}}}{\overset{︷}{\log\left(\beta^{t+1}\left(x_{j}^{t+1}\right)\right)+\log\left(p\left(x_{i}^{t}|x_{j}^{t+1}\right)\right)+\log\left(p\left(I^{t+1}|x_{j}^{t+1}\right)\right)}}\right)\right)$$

(15) $$\log\left(\beta^{t}\left(x_{i}^{t}\right)\right)=\log\left(\sum_{j=1}^{N}\exp\left(\log\left(\beta^{t+1}\left(x_{j}^{t+1}\right)\right)+\log\left(p\left(x_{i}^{t}|x_{j}^{t+1}\right)\right)+\log\left(p\left(I^{t+1}|x_{j}^{t+1}\right)\right)-b_{\max}\right)\right)+b_{\max}$$

Finally, the logarithm of the marginal probability is given by

(16) $$\log\left(p\left(x_{i}^{t}|I^{1:T}\right)\right)\propto \log\left(\alpha^{t}\left(x_{i}^{t}\right)\right)+\log\left(\beta^{t}\left(x_{i}^{t}\right)\right)$$

and, as discussed earlier, this probability is calculated for each n=1,…,N. An optimal bipartite assignment for each frame *t* is then achieved by applying the Hungarian algorithm to minimize an N×N matrix of costs given by

(17) $$\left(\begin{array}{cccc} -\log(p\left(x_{1}^{t}\to 1|I^{1:T}\right)) & -\log(p\left(x_{2}^{t}\to 1|I^{1:T}\right)) & \cdots & -\log(p\left(x_{N}^{t}\to 1|I^{1:T}\right))\\ -\log(p\left(x_{1}^{t}\to 2|I^{1:T}\right)) & -\log(p\left(x_{2}^{t}\to 2|I^{1:T}\right)) & \cdots & -\log(p\left(x_{N}^{t}\to 2|I^{1:T}\right))\\ \vdots & \vdots & \ddots & \vdots \\ -\log(p\left(x_{1}^{t}\to N|I^{1:T}\right)) & -\log(p\left(x_{2}^{t}\to N|I^{1:T}\right)) & \cdots & -\log(p\left(x_{N}^{t}\to N|I^{1:T}\right)) \end{array}\right).$$

The output of the assignment is an ordered set of detections, denoted $\left\{{\hat{x}}_{1:N}^{1}\right\},\ldots ,\left\{{\hat{x}}_{1:N}^{T}\right\}$.

## 4. Training Details and Evaluation Methodology

Tracking performance is evaluated on a collection of videos by comparing the system outputs to human-annotations, where both the shoulder-tail location and ear tag ID are provided for each animal in each frame. The following three scenarios are considered in the evaluation.

1. **Location**: The user is only interested in the location/orientation of each animal and the specific ID can be ignored. This scenario applies when only pen-level metrics are desired, such as average distance traveled per animal or pen space utilization.
2. **Location and ID (Initialized)**: Both the location/orientation and the ID of each animal are desired and the human annotations are provided for the first frame. This scenario assumes that several videos are being processed in sequence and that tracking results from the previous video are available. Location/orientation with ID are important for individualized metrics, such as monitoring health and identifying aggressors.
3. **Location and ID (Uninitialized)**: This scenario is the same as Location and ID (Initialized), except that human annotations are not provided for the first frame. This is the most challenging scenario because it forces the method to visually ID each animal from intermittent views of the ear tags within the time span of the video.

The method described in Section 3 is evaluated according to each of these scenarios in Section 5.

In the following, network training used to convert ear tag views into likelihood vectors is described in Section 4.1. Then, the dataset used for evaluation is described in detail in Section 4.2 and the metrics used for tracking success and failure are defined in Section 4.3.

### 4.1. Ear Tag Classification

**Figure 8:**
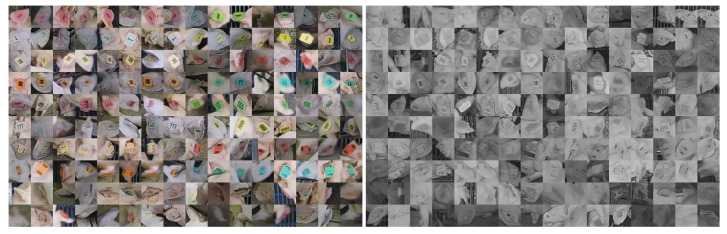
Samples of each tag crops used to train the deep classification network. The left image depicts crops taken from daytime color images and the right image depicts crops taken from nighttime infrared images. The first eight rows provide samples of each of the 16 tag types. The last two rows illustrate samples from the unknown tag ID category.

The proposed method identifies both the location and ID of each pig via separate networks. The dataset used to train the detector was introduced and provided by [28]. A set of 13,612 cropped color images of ear tag locations were used to train a classification network. A separate network was trained for grayscale (infrared) images using 6819 cropped images. The crops were labeled via human annotated as either belonging to one of the 16 known ear tags or to a category of “unknown tag ID.” When a tag image is classified as unknown tag ID, its target likelihood vector for training is set to $\frac{1}{16}$ for all categories. Figure 8 provides eight samples of each tag category along with 32 examples of unknown tag ID for both color and grayscale images.

Ear tag classification training was done using stochastic gradient decent with momentum (0.9). It is important to note that, while the output is passed through a softmax layer to ensure a valid probability vector, training is done with MSE regression on the outputs. This allows for the network to target both one-hot vectors and uniform probabilities.

### 4.2. Dataset Description

To evaluate the proposed tracking method, a human-annotated dataset was created. The data, along with cropped ear tag images and their corresponding categorizations, is available for download at http://psrg.unl.edu/Projects/Details/12-Animal-Tracking. It contains a total of 15 videos, each of which is 30 min in duration. The resolution of the videos is 2688 × 1520 and each was captured and annotated at 5 frames per second (fps). This frame rate was chosen empirically because it was deemed the minimum rate at which a human observer could comfortably interpret and annotate the video, keeping up with nearly all kinds of movement in the pen environment. Higher frame rates are nearly always better for tracking, but they come at the expense of increased processing times and, after a certain point, the improvements to tracking become negligible.

**Figure 9:**
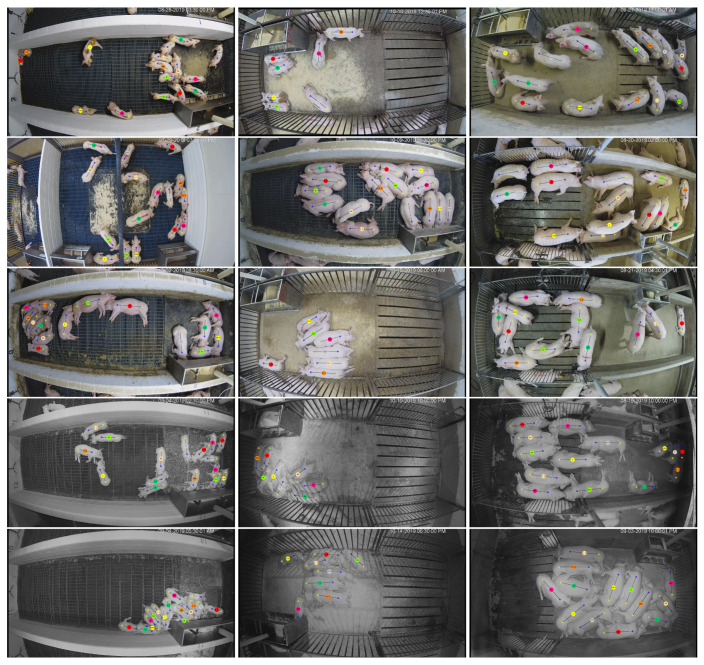
The first frames of the fifteen videos used to evaluate tracking performance. Annotations over each pig illustrate the position of the pig’s tail and shoulder along with the tag ID that each pig is equipped with (located between the shoulder and tail locations). The first column represents videos of the nursery phase (3–10 weeks old), the middle column represents the early finisher phase (11–18 weeks old), and the last column represents the late finisher phase (19–26 weeks old). The five rows correspond to high activity during the day, medium activity during the day, low activity during the day, medium activity during the night, and low activity during the night.

The videos depict different environments, numbers of pigs, ages of pigs, and lighting conditions. Table 1 summarizes the videos and their properties. Figure 9 shows the first frame of each video with each pig’s shoulder, tail, and ID illustrated via annotation. Note that annotations are provided for every frame of the video, but only the first frame is show here.

### 4.3. Performance and Analysis

To analyze tracking performance, a matched detection and a missed detection must be defined. Unlike many tracking applications, the number of targets in the field of view remains constant in group-housing livestock facilities and the ground truth position of the head and tail of each target is provided in each frame. Furthermore, it is assumed that the tracker knows how many targets are in the environment, so the number of detections provided by the tracker and the number of targets in the scene are always equal. Let $\left\{{\hat{x}}_{1:N}^{1}\right\},\ldots ,\left\{{\hat{x}}_{1:N}^{T}\right\}$ be the collection of *N* shoulder-tail pixel coordinates for *T* frames of a video sequence provided by a tracking algorithm, and let $\left\{{\bar{x}}_{1:N}^{1}\right\},\ldots ,\left\{{\bar{x}}_{1:N}^{T}\right\}$ denote the corresponding ground truth human annotations. The distance between the predicted target *i*’s position and the actual position of target *i* in frame *t* is defined as

(18) $$\Delta \left({\bar{x}}_{i}^{t},{\hat{x}}_{i}^{t}\right)=|{\bar{s}}_{i}^{t}-{\hat{s}}_{i}^{t}\left|+\right|{\bar{t}}_{i}^{t}-{\hat{t}}_{i}^{t}|$$

and the length of the ground truth target from shoulder to tail is

(19) $$\ell \left({\bar{x}}_{i}^{t}\right)=\left|{\bar{s}}_{i}^{t}-{\bar{t}}_{i}^{t}\right|.$$

Given these two definitions, successful matching events are defined as follows.

- Successful Match (Location and ID):
  $i=\underset{j=1,\ldots ,N}{\mathrm{argmin}}\Delta \left({\bar{x}}_{j}^{t},{\hat{x}}_{i}^{t}\right)$ and $i=\underset{j=1,\ldots ,N}{\mathrm{argmin}}\Delta \left({\bar{x}}_{i}^{t},{\hat{x}}_{j}^{t}\right)$ and $\Delta \left({\bar{x}}_{i}^{t},{\bar{x}}_{i}^{t}\right)<\ell \left({\bar{x}}_{i}^{t}\right)$
- Successful Match (Location):
  $k=\underset{j=1,\ldots ,N}{\mathrm{argmin}}\Delta \left({\bar{x}}_{j}^{t},{\hat{x}}_{i}^{t}\right)$ and $i=\underset{j=1,\ldots ,N}{\mathrm{argmin}}\Delta \left({\bar{x}}_{k}^{t},{\hat{x}}_{j}^{t}\right)$ and $\Delta \left({\bar{x}}_{i}^{t},{\bar{x}}_{k}^{t}\right)<\ell \left({\bar{x}}_{i}^{t}\right)$

The first condition states that detection *i* must be closest to ground truth *i* and vice versa, while the sum of the shoulder-to-shoulder and tail-to-tail distances must not exceed the shoulder-to-tail distance of the ground truth. This distance, while heuristic, adapts to pigs of any size and ensures that the detected and ground truth locations are a plausible match. The second condition is less strict than the first. It imposes a back-and-forth matching criteria that requires that the minimum-distance match for the detection is also the minimum-distance match for the ground truth, but their indices (tag IDs) do not need to coincide.

## 5. Results

The results of the proposed tracking method after being evaluated using the dataset are provided in Table 2. It is worth noting that, because the number of targets is known to the detector and each target’s location is approximated in each frame, the number of false positives and false negatives is equal. Thus, precision and recall are the same.

**Figure 10:**
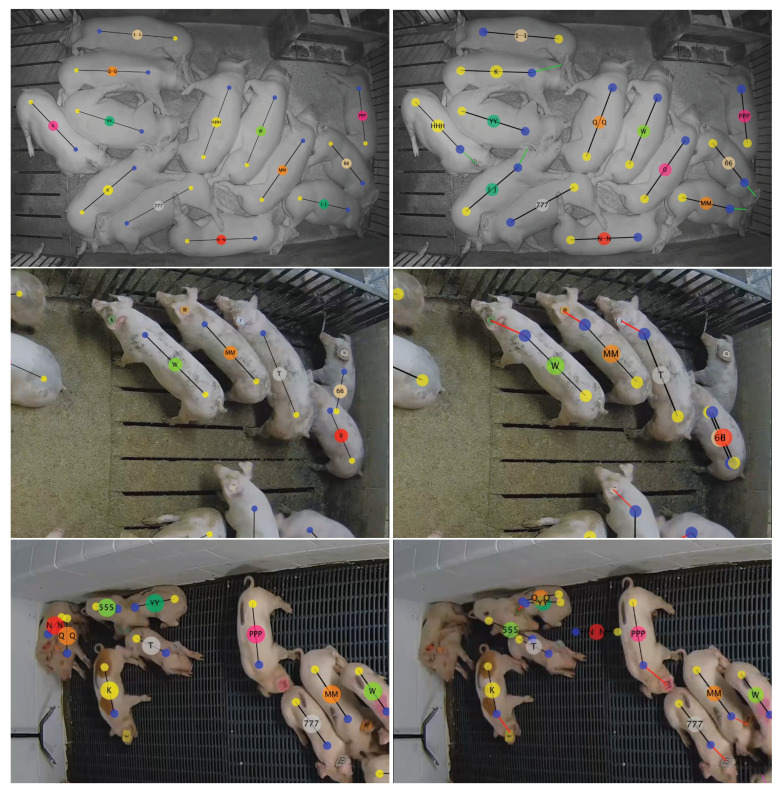
Failure cases for the proposed method in three different scenarios. The left images contain ground truth annotations and the right images are visualizations of the output of the proposed method.

As anticipated, the worst performance occurs when the locations and IDs of each pig are uninitialized, with an average precision/recall is 0.8251. This situation forces the method to infer the ID of each animal from glimpses of their ear tags within the 30-min duration of the video. The “Late Finisher: Low (Night)” video has the worst performance, at 0.5252 precision/recall. Figure 10 illustrates the ground truth and network output for several error examples, and the top one shows the first frame of the “Late Finisher: Low (Night)” video. Only seven of the 13 pigs are labeled with the correct ID, even though all 13 are detected and oriented correctly. This video is particularly challenging for ear tag classification because, in addition to being at night when ear tags are already more difficult to discern, half of the pigs do not significantly change position during the 30 min record time. Therefore, ear tag presentations are not varied enough to confidently identify each individual pig. It’s worth noting that, in an actual deployment of the system where multiple 30 min segments are processed in sequence, there is a good chance that the ear tags will be viewed and classified in preceding videos. The “uninitialized” assumption is really a worst case scenario that ignores prior observations.

The second row of Figure 10 illustrates a different kind of error. The pig labeled ‘66’ is sitting in the corner of the pen and its tail area is occluded by pig ‘II’. Pig ‘II’ also has its head occluded and the method, at some point earlier, detected a pig with reversed shoulder and tail at the same location at ‘II’. This detection likely occurred when ‘66’ was partially occluded and the method assigned the erroneous detection to the ‘66’ ID. In general, occlusions cause missed detections (false negatives) and the method is susceptible to mistaking the shoulders for the tail area when the pig’s head is down toward the ground and not visible to the camera.

Errors in the third row of Figure 10 illustrate a situation where multiple targets are not detected for long enough periods of time that the method holds their last observed location until they are re-identified. This occurred for two reasons. First, pigs viewed from the side are more prone to occlusion than pigs viewed from a top-down perspective. Second, targets are smaller in this view so the detection network has less pixels and, correspondingly, less features per target. This could be at least partially corrected by processing larger images, but this would come at the expense of longer processing times.

### Hardware and Processing Times

The method was implemented in MATLAB using the Deep Learning Toolbox. The desktop computer used to process the videos has an Intel i9-9900K 8-core CPU, 32 GB of DDR4 RAM, 512 GB of m.2 SSD memory, and an NVIDIA RTX2080ti GPU. Before processing frames with the fully-convolutional detector, they are downsampled to a resolution of 576×1024×3 (rows × columns × channels), and 24 frames are stacked together before processing on the GPU. It takes the computer ≈0.5 s to process the batch of 24 images. To classify ear tags, all ear tag windows are gathered together into a large batch of 64×64×3 images and processed all-at-once by the classification network. Classification takes, on average, 0.2 s for 24 images. All other processes involved in detection, including reading video frames and down-sampling, consume an additional 0.7 s per batch of 24 images. Thus, detection and ear tag classification take approximately 0.054 s per frame (18.5 fps).

The proposed multi-object tracking method using fixed-cardinality interpolation and forward-backward inference takes 20 s to process a 30-min video with 16 pigs and this time drops to 6 s with 7 pigs. Fixed-cardinality interpolation consumes approximately 75% of that time and forward-backward inference uses the remaining 25%. The computational complexity of fixed-cardinality interpolation is $O(TN^{3})$, where *T* is the number of frames and *N* is the number of targets. This is due to the fact that the Hungarian algorithm, with complexity $O(N^{3})$, is used to associate every pair of neighboring frames. In practice, with 16 targets, this adds 0.01 s per frame and brings the total to 0.064 s per frame (15.6 fps). The videos used to analyze the method were recorded at 5 fps, so this performance demonstrates that video can comfortably be processed in real-time.

## 6. Conclusions

This paper presents a method for long-term tracking of individual livestock in group-house settings. The method takes advantage of the power of deep convolutional neural networks to detect individual targets and classify their identities. A probabilistic framework is used to efficiently combine per-frame detection and classification across long frame sequences.

The publicly-available, human-annotated dataset introduced in this work can be used to evaluate performance for long-term tracking of group-housed livestock. By representing a variety of different environments, ages/sizes of animals, activity levels, and lighting conditions, the dataset exposes the strengths and weaknesses of tracking methods. Results demonstrate that the method achieves an average precision and recall greater than 0.9 across a variety of challenging scenarios. While this work focuses on pigs, it is expected that the underlying techniques could easily be adopted to a variety of other livestock animals.

This location and orientation tracking method could be used as the foundation for a more sophisticated tracker of activity and behavior. In terms of extracting activities, it would be relatively straight-forward to convert the image-space tracking outputs to pen-space distance traveled using known camera parameters and pose estimation to the pen space. Eating, drinking, and social interactions can be approximated from proximity of targets to fixed landmarks and other targets.

In this work, industry-standard ear tags were used for visual identification. Ideally, long-term tracking of individuals could be achieved without augmenting targets. However, the homogeneity of livestock populations makes it difficult to discern differences between individuals. Preliminary work suggests that this might be possible using facial recognition [71], but applications to long-term tracking are untested and facial recognition would likely require addition cameras in the pen space to get close-up shots.

## Figures and Tables

**Table 1:** Properties of the fifteen videos captured and annotated for tracking performance analysis. For each age range (nursery, early finisher, and late finisher), three videos were captured during the day with the lights on and two videos were captured at night using IR video capture and IR flood lights to illuminate the scene. The activity levels for the pigs were subjectively categorized as either High (H), Medium (M), or Low (L).

	Nursery					Early Finisher					Late Finisher				
Video #	1	2	3	4	5	6	7	8	9	10	11	12	13	14	15
Day	X	X	X			X	X	X			X	X	X		
Night				X	X				X	X				X	X
# of Pigs	16	16	15	16	16	7	15	7	8	8	16	14	12	14	13
Activity Level	H	M	L	M	L	H	M	L	M	L	H	M	L	M	L

**Table 2:** Precision/recall results for all 15 videos in the human-annotated dataset. The precision/recall results in “Location” do not require the tracker to provide the correct ID for animals. Instead, it is only required that each animal’s location is matched with a detection. The “Location and ID” results require the tracker to correctly identify the location and correct ID of a pig in order to be counted as a true positive. The “(Uninitialized)” variant does not provide the location and ID of each pig in the first frame, whereas the “(Initialized)” variant does.

Location							
	Activity	High (Day)	Medium (Day)	Low (Day)	Medium (Night)	Low (Night)	Average
Age							
Nursery		0.9267	0.9964	0.9985	0.9548	0.8405	**0.9434**
Early Finisher		0.9961	0.9973	1	0.9349	1	**0.9857**
Late Finisher		0.9907	0.989	0.9969	0.9564	1	**0.9866**
**Average**		**0.9711**	**0.9943**	**0.9984**	**0.9487**	**0.9468**	**0.9719**
**Location and ID (Initialized)**							
	**Activity**	**High (Day)**	**Medium (Day)**	**Low (Day)**	**Medium (Night)**	**Low (Night)**	**Average**
**Age**							
Nursery		0.8893	0.9941	0.9933	0.8958	0.6256	**0.8796**
Early Finisher		0.9949	0.9847	1	0.8716	1	**0.9702**
Late Finisher		0.9836	0.958	0.9897	0.8569	0.8462	**0.9269**
**Average**		**0.9559**	**0.9789**	**0.9943**	**0.8748**	**0.8239**	**0.9256**
**Location and ID (Uninitialized)**							
	**Activity**	**High (Day)**	**Medium (Day)**	**Low (Day)**	**Medium (Night)**	**Low (Night)**	**Average**
**Age**							
Nursery		0.8893	0.9941	0.694	0.7927	0.6108	**0.7962**
Early Finisher		0.9948	0.9718	1	0.8946	0.5888	**0.89**
Late Finisher		0.9836	0.8176	0.9897	0.629	0.5252	**0.789**
**Average**		**0.9559**	**0.9278**	**0.8946**	**0.7721**	**0.5749**	**0.8251**
